# Polychromatic polarization microscope: bringing colors to a colorless world

**DOI:** 10.1038/srep17340

**Published:** 2015-11-27

**Authors:** Michael Shribak

**Affiliations:** 1 Marine Biological Laboratory, 7 MBL St, Woods Hole, MA 02543, USA

## Abstract

Interference of two combined white light beams produces Newton colors if one of the beams is retarded relative to the other by from 400 nm to 2000 nm. In this case the corresponding interfering spectral components are added as two scalars at the beam combination. If the retardance is below 400 nm the two-beam interference produces grey shades only. The interference colors are widely used for analyzing birefringent samples in mineralogy. However, many of biological structures have retardance <100 nm. Therefore, cells and tissues under a regular polarization microscope are seen as grey image, which contrast disappears at certain orientations. Here we are proposing for the first time using vector interference of polarized light in which the full spectrum colors are created at retardance of several nanometers, with the hue determined by orientation of the birefringent structure. The previously colorless birefringent images of organelles, cells, and tissues become vividly colored. This approach can open up new possibilities for the study of biological specimens with weak birefringent structures, diagnosing various diseases, imaging low birefringent crystals, and creating new methods for controlling colors of the light beam.

Natural white light consists of a mixture of monochromatic waves with wavelengths ranging from 380 nm to 700 nm^1^. If the light beam is spilt into two parts and then recombined, we can observe interference. Each monochromatic wave produces its own interference pattern. Some waves experience destructive interference and their intensity is diminished. The intensity of other waves increases due to constructive interference. The combined beam exhibits the Newton’s interference colors. The hue is determined by the wavelength, which is missing from the spectrum due to destructive interference.

There are two types of interference colors, one with initial zero phase shift between two interfering beams (white achromatic fringe, constructive interference) and another with initial half-wave phase shift (black achromatic fringe, destructive interference)^2^,^3^,^4^,^5^. They produce complementary color sequences, which are described by the Newton’s scale of color. The second type of interference colors appears during reflection from a soap bubble, two close spherical and flat glass surfaces (Newton color rings), oil slick in a puddle or oil spot on wet asphalt, etc. This type of interference is employed in interference and polarization microscopy because it is more sensitive for low retardance change and carries less shot noise. For small retardance (<200 nm), the destructive interference is relaxed for all wavelengths simultaneously and the brightness of the region increases, first with a white spectral composition. But after the retardance approaches 400 nm, the blue part of spectrum is suppressed and the specimen become yellow and then red. Once the retardance reaches 600 nm, the red part of spectrum is blocked out and the specimen turns to blue and then green. The color changes in this sequence three more times until the retardance reaches 2000 nm. Then the interference colors turn white and the retardance can no longer be reliably determined using the region’s spectral composition. The polarized light coloring has been widely used in mineralogy and petrography for many years^6^,^7^,^8^,^9^,^10^,^11^,^12^. But this phenomenon previously could not be utilized in biology because many biological specimens exhibit retardance of several tens of nm or less and therefore are colorless.

We developed a new polychromatic polarized light microscope (polychromatic polscope) that produces interference colors at retardance of several nm. Traditional Newton colors require that the interfering beams with the same polarization states, and the beam amplitudes are added as two scalars. In our approach of generating interference colors we utilize the beam polarization and amplitudes of the interfering beams, which are added as two vectors. In polychromatic polscope the hue is determined by the orientation of the birefringent structure, not by its retardance. Thus, full spectrum color can be achieved at a much lower retardance. The polychromatic polscope shows the orientation-independent birefringence image without requiring any digital computation. An eye or camera can directly see the colored polarization image in real time through the ocular with brightness corresponding to retardance and color corresponding to the slow axis orientation. Previously colorless organelles, cells, and tissues birefringent images become vividly colored.

## Results

### Polychromatic polscope

The optical design of polychromatic polscope is based on a standard polarized light microscope equipped with special spectral polarization state generator and analyzer. The specimen under investigation is illuminated by white light with polarization depended on the wavelength. The specimen modifies the beam polarization. The analyzer could be either orthogonal or parallel to the polarization state generator. In the first case the analyzer blocks polarization that is unmodified by the specimen. Thus, non-birefringent specimen parts are black and the birefringent structures are colored. In the second configuration the analyzer reduces equally all spectral polarization states, created by the polarization generator. In this case non-birefringent area appears is gray (neutral). Also colors of the birefringent structures are more intense and non-birefringent features are still visible in gray.

**Figure 1:**
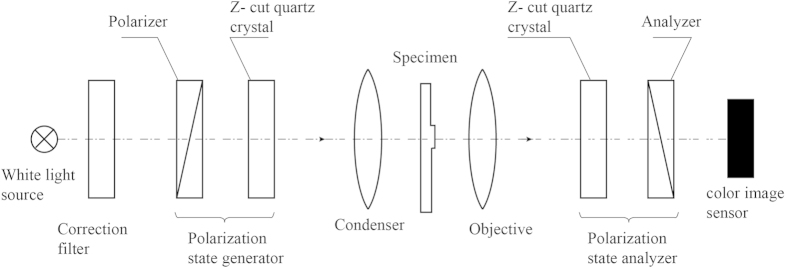
Schematic of polychromatic polarization microscope (polychromatic polscope).

An example of the polychromatic polscope is shown in Fig. 1. It consists of a white light source with optional bandpass filter, polychromatic polarization state generator, condenser lens, specimen under investigation, objective lens, polychromatic polarization state analyzer, and color image sensor. The polychromatic polarization state generator is formed by the linear polarizer with orientation *ψ*, and a Z-cut quartz crystal, whose optical axis is perpendicular to the window surface. The polychromatic polarization state analyzer consists of another Z-cut quartz crystal and linear analyzer with orientation *ψ* + 90°.

The polychromatic polscope works in the following way. The spectrum of beam, radiated by the light source, is shaped by a bandpass filter. The filter can be placed in any location in the light beam path. Instead of the broadband light source one can use several monochromatic light sources, for example lasers. In this case the bandpass filter is not needed. The filter is also not required if the spectrum of the beam is effectively restricted by the detector spectral sensitivity. For example, the radiation spectrum of the Sun or an incandescent bulb is effectively restricted by human eye sensitivity from about 400 nm to about 700 nm. The polarizer produces a linear polarized beam with polarization oriented at *ψ*. Then the first Z-cut quartz crystal rotates the beam polarization plane by some angle *ϕ*, which depends on the wavelength *λ*. Thus, orientation of the electric vectors after the polychromatic polarization state generator becomes spectrally dispersed.

In principle, other optical active media can be employed in order to create the required spectral polarization distribution instead of quartz. In all well-authenticated cases of optical activity, the crystal or molecular structure is of the type which can exist in two enantiomorphous forms, i.e. forms which are related to one another as an object and its mirror image, but do not differ in any other way. In one form the spirals for a given direction are right-handed and in the other left-handed, and their specific rotations are the same numerically but have opposite sign. In crystals, the amount of rotation of light for a specific wavelength for a one millimeter thickness of a plate is the specific rotation, and is either right-handed (dextrorotatory) or left-handed (levorotatory) as viewed looking toward the light source. For liquids it is common practice to measure the specific rotation in a column 10 cm in length. Lists of minerals and inorganic and organic compounds that are known to be optically active have been tabulated in many places^2^,^7^.

Let us describe polarization transformation by the polychromatic polarization state generator in more detail. The polarizer produces linear polarized beam with orientation *ψ*. Then the beam passes through the optical active polarization rotator, which rotates its polarization by angle *ϕ* without introducing ellipticity^13^:


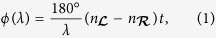


where *t* is thickness of the polarization rotator, *λ* is wavelength, *n*_*L*_ and *n*_*R*_ are refractive indices of the left and right circular polarizations, respectively. If *n*_*L*_ > *n*_*R*_ then the polarization rotator is *d*-rotatory, and if *n*_*R*_ > *n*_*L*_ then the polarization rotator is *l*-rotatory.

In the selected spectral domain the minimal and maximal polarization rotation angles *ϕ*_min_ and *ϕ*_max_ correspond to the longest and shortest wavelengths *λ*_max_ and *λ*_min_, respectively:


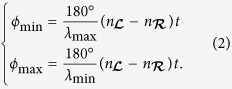


The difference between maximal and minimal polarization rotations is 90°. In many cases the spectral dispersion of circular birefringence *n*_*L*_ - *n*_*R*_ is low and we can approximately assume that it does not depend on the wavelength. Then thickness *t* of the polarization rotator can be found using the following formula:


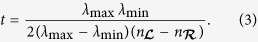


After substituting (3) into equation (1) and taking into account the 1^st^ equation (2) we obtain the spectral dependence of polarization plane orientation of the illuminating beam:


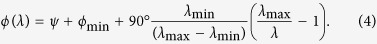


It is convenient to choose orientation of the polarizer *ψ* = −*ϕ*_*min*_. Then equation (4) is simplified:


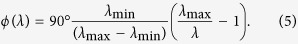


In particular, for the visible spectrum from 440 nm to 660 nm we have the following spectral dependence of polarization plane orientation:


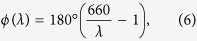


where wavelength *λ* is in nanometers.

As one can see, the polarization plane of red spectral component (*λ* = 660 nm) is parallel to the initial axis (*ϕ* = 0°). The polarization planes of orange (*λ* = 609 nm), yellow (*λ* = 566 nm), green (*λ* = 528 nm), cyan (*λ* = 495 nm) and blue (*λ* = 466 nm) components are oriented at angles 15°, 30°, 45°, 60° and 75° to the initial axis, correspondently.

The second Z-cut quartz crystal introduces the inverse polarization rotation and inverse spectral dispersion. Thus, all electric vectors of the beam become oriented at *ψ*. The analyzer, which is oriented at *ψ* + 90°, extinguishes the beam. The optical configuration shown in Fig. 1 works as the crossed linear polarizer and analyzer with the principal plane orientation *ϕ*(*λ*) (see eq. (5)).

The intensity of light ***I***(*λ*), which is transmitted by a birefringent specimen between crossed linear polarizer and analyzer, is described by formula (see, for example^14^):


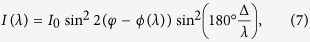


where ***I***_***0***_ is intensity of the beam after the polarizer, *φ* and *Δ* are slow axis orientation and retardance (in nm) of the specimen. For simplicity we assume that the extinction factor^15^,^16^ is high enough and therefore we do not take into account the depolarization and scattered light.

A spectral component with wavelength *λ*_*ext*_ is extinguished (***I***(*λ*_*ext*_) = 0) when *φ* *=* *ϕ*(*λ*) or *Δ* *=* *mλ*, where *m* is an integer (*m* = 0, 1, 2…). If the specimen retardance is less than the minimum wavelength in the used spectral band (*Δ* *<* *λ*_min_) then extinguished wavelength depends on the specimen slow axis orientation only:


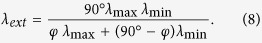


As one can see if the slow axis is parallel to the initial axis (*φ* = 0°) then *λ*_*ext*_ = *λ*_max_, if *φ* = 90° then *λ*_*ext*_ = *λ*_min_. Let’s assume that the slow axis of the birefringent specimen under investigation is oriented at 30°. Then *λ*_*ext*_ = 566 nm. The slow axis is parallel to electric vector of the yellow polarization component and oriented at 45° to electric vector of the blue component. In this case, the yellow component passes through the specimen without change of polarization and ellipticity of the blue component is increased. The analyzer extinguishes the yellow color and the specimen appears in blue. If the specimen is turned by 45° then *φ* = 75° and *λ*_*ext*_ = 466 nm. The specimen’s retardance increases ellipticity of the yellow component and does not affect the blue electric vector. The analyzer removes the blue color and the specimen appears in yellow. The empty background area does not affect the beam ellipticity and therefore it appears dark. The image brightness reflects amount of retardation, and the image color depicts the slow axis orientation. If the specimen retardance is large (*Δ* *≥* *λ*_min_) the extinguished wavelength depends on the both slow axis orientation and retardance. The color picture becomes more complicated.

In a case of classical Newton interference (*ϕ*(*λ*) = 0°) the spectral extinction occurs at *φ* = 0° for all wavelength simultaneously. At this orientation the specimen is dark without any coloring independently on its retardation. The image contrast disappears. The intensity is maximal when the slow axis is oriented at 45°. The first extinguished wavelength occurs at *λ*_min_. The color is determined by specimen retardance only and it does not depend on slow axis orientation.

Many variations of polychromatic polscope design are possible and already some have been implemented. It can be used in transmitted or reflected light. In the latter case, a half mirror separates the illuminated and reflected beams. The polychromatic polarization state generator and analyzer can be placed in the reverse order in the both transmitted and reflected light schemes.

### Imaging diatom *Arachnoidiscus*

Diatom *Arachnoidiscus* is an excellent specimen for demonstrating advantages of the polychromatic polscope. It has silicified cell wall, which forms a radially symmetric pillbox-like shell (frustule) composed of overlapping halves that contain intricate and delicate patterns. Sometime it is called “a wheel of glass”. Diatom *Arachnoidiscus* deserves the term of “living photonic crystals” and was employed for enabling sub-diffractive focusing with better confining of the light beam than other far-field super focusing approaches^17^. Birefringence of the *Arachnoidiscus* structure is very low, except the central radial filaments that exhibit slightly elevated retardance. According to our previous measurement^18^, retardance of the central filaments is about 5 nm.

**Figure 2:**
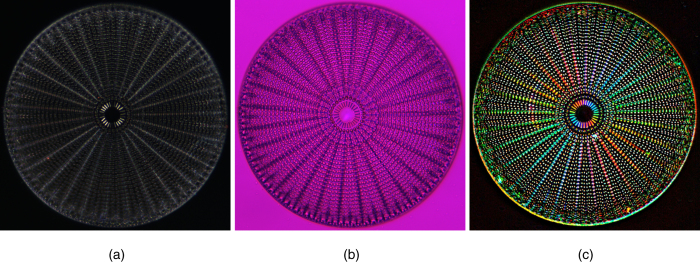
Images of a diatom *Arachnoidiscus*. All images were taken in white light. The picture size is 190 μm × 190 μm. The maximum retardance is 5 nm. The left photo (**a**) depicts a case with crossed linear polarizer and analyzer. The middle image (**b**) illustrates a case with full-wave plate inserted into the optical path. The right picture (**c**) shows polarization polychromatic image with the brightness corresponding to retardance and hue representing the principal (birefringent) axis orientation.

Three color images of the diatom *Arachnoidiscus* were taken with various polarized light techniques are shown in Fig. 2. The left picture (Fig. 2a) was captured with crossed polarizer and analyzer. The central radial filaments are slightly brighter at the diagonal directions. Vertical and horizontal filaments are dark.

We can also shift the Newton colors by adding a full-wave plate (retardance ~550 nm), which is also known as “sensitive” or “red” waveplate^19^. The middle picture (Fig. 2b) illustrates the result with crossed polarizer and analyzer and added full-wave plate. The entire image is purple with a hardly visible hue change in the central diagonal filaments.

The right picture (Fig. 2c) was taken with polychromatic polscope. It shows the diatom image after background subtraction. The brightness corresponds to retardance and hue represents the slow axis orientation. The colors confirm that birefringent structure of diatom has radially oriented principal axes.

### Imaging bdelloid rotifer *Adineta vaga*

The polychromatic light polarization technique allows to see a colored polarization image directly and capture the picture instantly, in real time. Therefore, it enables to achieve the highest temporal resolution. As shown, the polychromatic polscope provides sharp images of fast-moving, low-birefringent structures and makes visible rapid processes accompanied by birefringence changes. In combination with a pulse light source, the new microscope should make it possible to visualize development of nerve pulses, shock wave propagation, etc.

**Figure 3:**
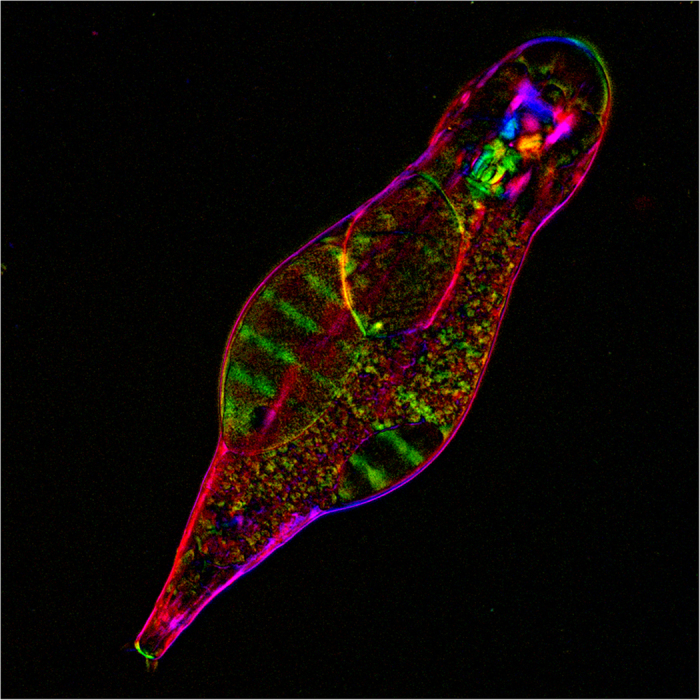
Polarization polychromatic image of bdelloid rotifer *Adineta vaga*. The picture size is 283 μm × 283 μm.

We successfully applied the polychromatic polarization technique for imaging various types of fast-moving bdelloid rotifers. Bdelloid rotifers are microscopic freshwater invertebrates best known for their capacity to undergo frequent cycles of desiccation and rehydration at any life stage, for their long-term asexuality, which is manifested in the absence of males and meiosis, and for the ability to capture foreign genetic material at levels unprecedented in metazoans^20^. A live image of the bdelloid rotifer *Adineta vaga* visible with polychromatic polarized light microscope under 20 x magnification is shown in Fig. 3. This image of an adult female emphasizes characteristic structures such as the pharynx with a mastax consisting of hard jaws (trophi), a set of circular and longitudinal muscles, bilateral ovaries with oocyte nuclei, and the parthenogenetically developing egg.

### Imaging malaria infected red blood cells

**Figure 4:**
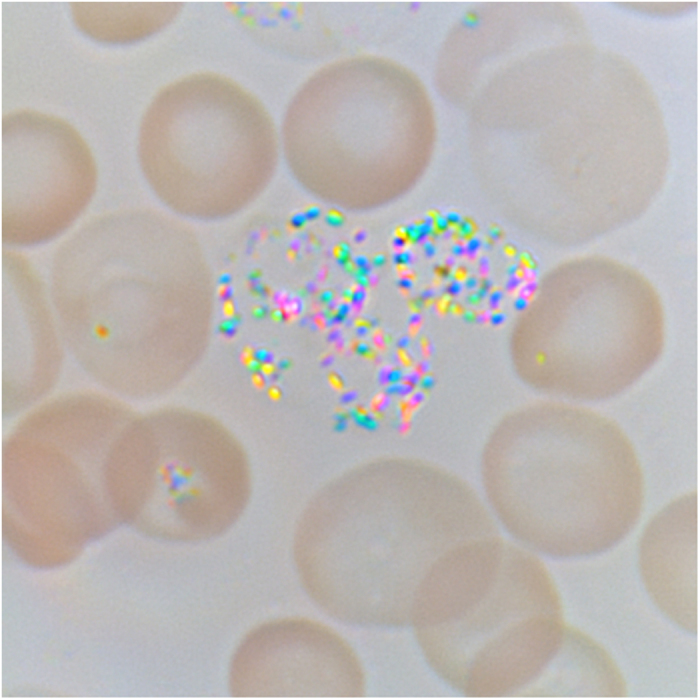
Polarization polychromatic image of blood smear from malaria infected mouse. The image size is 26 μm x 26 μm.

Polarized light microscopy has been used for malaria study for a long time^21^,^22^,^23^. It allows to see malarial pigment, hemozoin, which is a crystalline product of the digestion of hemoglobin by the parasites. By imaging hemozoin directly, polarization microscopy does not require exogenous contrast agents to diagnose malaria. It can be easy to use for analysis of fresh samples. We examined Plasmodium yoelii infected mice, fixed and unfixed. Polychromatic image of blood smear is shown in Fig. 4. Colored brilliantly birefringent granules of the hemozoin are clear visible. The small hemozoin granules are surrounded by large brownish red blood cells. In a fresh sample, the hemozoin granules freely rotate and change the color according to their optical axis orientation. The hemozoin appears as appealing Christmas lights that are blinking in changing colors.

### Imaging collagenous stroma in cancer tissue

Polychromatic polscope allows one to visualize collagen without need of special stains or second-harmonic-generation microscopy. Collagen comprises almost one quarter of the protein in the human body. Understanding how collagen is organized can lead to an understanding of how to restore healthy tissue to areas affected by burns, scarring, diseases, etc. The polychromatic technique can be useful in quantifying the degree of build-up in atherosclerotic plaques, identifying collagen organization in fibrosis, and measuring the progression of synthetic tissue in tissue engineering. Collagen is revealed with high sensitivity with standard hematoxylin and eosin (H&E) stained sections.

**Figure 5:**
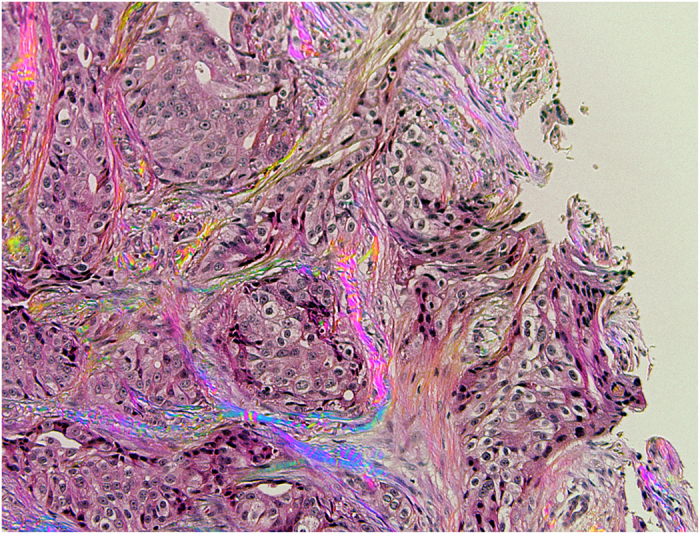
Polarization polychromatic image of birefringent collagen fibers in H&E-stained breast cancer tissue. The picture size is 660 μm × 867 μm.

Figure 5 shows an example of polarization polychromatic image of a histological specimen with dense tumors and invasion into the collagenous stroma around them. The specimen is stained by H&E. In the picture the variable color swirling collagenous stroma surrounds the nests of pink cancer cells. The stroma brightness reflects amount of retardation of the collagen fibers, and the color depicts their slow axis orientation.

### Imaging mouse brain section

**Figure 6:**
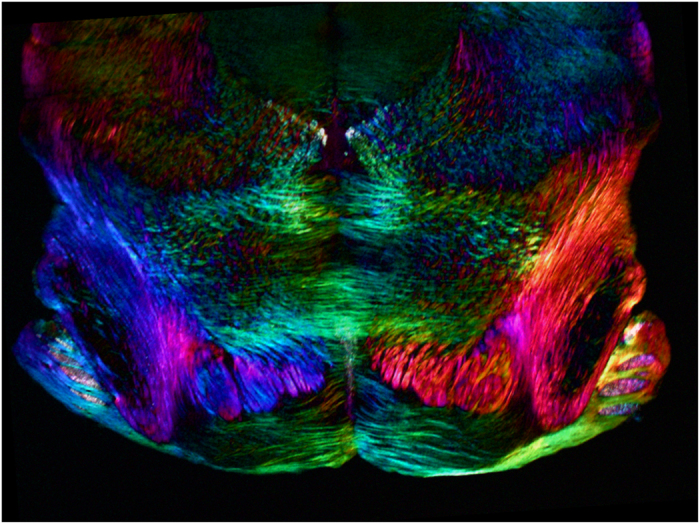
Polarization polychromatic image of mouse brain slice. The picture size is 2.9 mm × 2.1 mm.

Polarized light microscopy has been extensively used in brain studies, for example, to examine wall structural integrity of brain arteries^24^, the paths of white matter fiber tracts in the human brain^25^,^26^,^27^,^28^,^29^, wall strength in saccular brain aneurysms^30^, unstained cochlear cross-sections of the mouse^31^, etc. We tested new polychromatic polscope for imaging superior colliculus in mouse brain section^32^. No chemical treatment and no staining were applied to the specimen. Figure 6 shows a coronal view of a 20 μm thick section of the brain, which has been sliced down a vertical axis to divide it into front and back. Nerve cells appear in the real colors that reveal orientation of the molecules. The medial nucleus of the trapezoid body are clear visible.

### Imaging birefringent nanostructures written by laser

**Figure 7:**
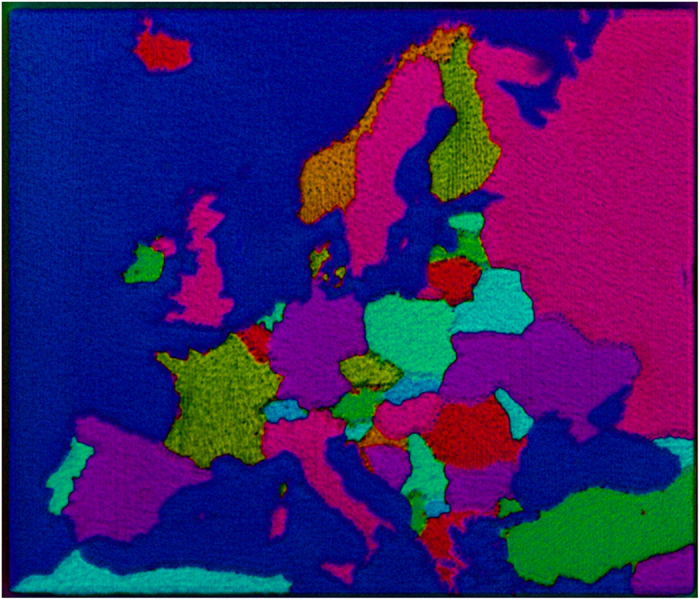
Polarization polychromatic image of femtosecond laser written map of Europe inside fused silica glass. The picture size is 196 × 167 μm.

Of course, using new polychromatic polscope is not restricted for biomedical applications. Figure 7 shows a real color image of the Europe map, which is created by a birefringent self-assembled nanostructure. The specimen was fabricated by laser nano-structuring technique developed by prof. Peter Kazansky group from Optoelectronics Research Centre at Southampton University^33^,^34^,^35^,^36^. The map of Europe was created with the femtosecond laser inside the fused silica substrate where territories of different countries were written with different polarization of the laser.

The written nanogratings manifest the form birefringence. Unlike intrinsic birefringence, which is due to the anisotropy of oriented molecules, the form birefringence is caused by alignment of submicroscopic assemblies of thin cylindrical rods or plane-parallel plates with refractive index different from the surrounding medium^37^,^38^. The plate assembly always behaves as a negative uniaxial crystal with its optical axis perpendicular to the plane of the plates. The rod assembly behaves as a positive uniaxial crystal with its optical axis parallel to the axes of the rods. For comparison, quartz crystal is a positive uniaxial crystal. The sign of the birefringence indicates whether the shape of the structural form is nearer to that of a rod or a plate. It has been shown that nanograting always acts as a negative uniaxial crystal^39^.

Two parameters of the birefringence, retardance and the slow axis orientation, can be independently controlled during the writing process. The slow axis is defined by the beam polarization. The retardance is a function of the laser fluence. Retardance depends also on the wavelength, the pulse duration and the number of laser pulses. The retardance can be controlled with a precision of about 10 nm, while slow axis angle can be defined with a few degrees precision^33^,^34^,^35^,^36^. Birefringent nanogratings can withstand temperatures of 1000°C, which makes them ideal for archiving a large volume of important information. The ability to record and read several layers of information via nanogratings was also demonstrated^36^.

## Discussion

We have developed the new polychromatic polscope that produces natural color images of birefringent structures with retardance <400 nm. Birefringent images in the real color are captured instantly. The contrast is generated at any specimen orientation. The polychromatic polscope can be employed to study collagen fibers in cancer tissue, crystalline hemozoin in malaria infected cells, brain structure, cilia in stentor, mastax, cilia and muscle fibers in rotifer, skeletal rods in Arbacia punctulata, muscle fibers and otholith in zebrafish, kidney stones, amyloid birefringence, etc.

## Methods

### Diatom *Arachnoidiscus* specimen

Diatom *Arachnoidiscus Ehrenbergi* microscope slide (Turtox slide series No. B1.144 “Diatoms”) was manufactured by General Biological Supply House, Inc. (Chicago, IL, USA). We used an upright light microscope Olympus BX61 (Olympus America, Center Valley, PA, USA) equipped with objective lens UPlanFl 40 x/0.75P and 100 W halogen lamp U-LH100-3-5. All images were taken in white light without any filter. The middle picture (Fig. 1b) was taken with adding a first order red, full-wave plate Olympus U-TP530. The images were captured with consumer SLR camera Nikon D40 x (Nikon, Melville, NY, USA). The image size is 190 μm × 190 μm.

### Bdelloid rotifer *Adineta vaga* specimen

Live bdelloid rotifer *Adineta vaga* specimen was provided by Irina Arkhipova of the Marine Biological Laboratory (Woods Hole, MA, USA). We used an upright light microscope Olympus BX61 (Olympus America, Center Valley, PA, USA) equipped with objective lens UPlanFl 20 x/0.50 P and 100 W halogen lamp U-LH100-3-5. The picture was taken in white light without any filter. The images were captured with Hamamatsu ORCA-3CCD camera C7780-20 (Hamamatsu Photonics, Japan). Exposure time was 5 ms. The image size is 283 μm x 283 μm.

### Plasmodium yoelii infected mouse blood smear

Microscope slide with blood smears of Plasmodium yoelii infected mouse was provided by Photini Sinnis of Johns Hopkins Malaria Research Institute (Baltimore, MD, USA). We used an inverted light microscope Olympus IX81 (Olympus America, Center Valley, PA, USA) equipped with objective lens UPlanFl N 100x/1.30 Oil P, 2 x zoom lens and 100 W halogen lamp U-LH100-3-5. The picture was taken in white light without any filter. The images were captured with Olympus CCD camera DP73. The image size is 26 μm x 26 μm.

### Cancer tissue with collagenous stroma

The H&E-stained breast cancer tissue with birefringent collagen fibers was provided by Richard Levenson of UC Davis Medical Center (Sacramento, CA, USA). We used an inverted light microscope Olympus IX81 (Olympus America, Center Valley, PA, USA) equipped with objective lens UPlanFL N 10 x/0.30 P and 100 W halogen lamp U-LH100-3-5. The picture was taken in white light without any filter. The images were captured with Hamamatsu ORCA-3CCD camera C7780-20 (Hamamatsu Photonics, Japan). The image size is 660 μm x 867 μm.

### Mouse brain section

Microscope slide with 20 μm thick section of mouse coronal brain stem was provided by Timothy Balmer of Georgia State University Neuroscience Institute (Atlanta, GA, USA). We used an inverted light microscope Olympus IX81 (Olympus America, Center Valley, PA, USA) equipped with objective lens UPlanFL 4 x/0.13, zoom lens 0.5 x and 100 W halogen lamp U-LH100-3-5. The picture was taken in white light without any filter. The images were captured with Olympus color CCD camera DP73. The image size is 2.9 mm x 2.1 mm.

### Europe map fabricated by laser nano-structuring technique

A fused silica glass slide with the Europe map was provided by Mindaugas Gecevicius and Martynas Beresna (University of Southampton, UK). We used an inverted light microscope Olympus IX81 (Olympus America, Center Valley, PA, USA) equipped with objective lens UPlanFL 20 x/0.50 P and 100 W halogen lamp U-LH100-3-5. The picture was taken in white light without any filter. The images were captured with Olympus color CCD camera DP73. The image size is 196 × 167 μm.

## Additional Information

**How to cite this article**: Shribak, M. Polychromatic polarization microscope: bringing colors to a colorless world. *Sci. Rep.* **5**, 17340; doi: 10.1038/srep17340 (2015).
